# Prognostic Value of High-Sensitivity Modified Glasgow Prognostic Score in Castration-Resistant Prostate Cancer Patients Who Received Docetaxel

**DOI:** 10.3390/cancers13040773

**Published:** 2021-02-12

**Authors:** Keisuke Ando, Shinichi Sakamoto, Shinpei Saito, Maihulan Maimaiti, Yusuke Imamura, Tomokazu Sazuka, Nobuo Sato, Akira Komiya, Naohiko Anzai, Tomohiko Ichikawa

**Affiliations:** 1 Department of Urology, Chiba University Graduate School of Medicine, Chiba 260-8670, Japan; yakuro3917@chiba-u.jp (K.A.); caua5660@chiba-u.jp (S.S.); you-i@wa3.so-net.ne.jp (Y.I.); acna4278@chiba-u.jp (T.S.); akirakomiya@mac.com (A.K.); tomohiko_ichikawa@faculty.chiba-u.jp (T.I.); 2 Department of Pathology, Chiba University Graduate School of Medicine, Chiba 260-8670, Japan; marghulan@chiba-u.jp; 3 Department of Urology, Funabashi Municipal Medical Center, Chiba 273-8588, Japan; gonta611223as@pa2.so-net.ne.jp; 4 Department of Pharmacology, Chiba University Graduate School of Medicine, Chiba 260-8670, Japan; anzai@chiba-u.jp

**Keywords:** Glasgow prognostic score, high-sensitivity modified Glasgow prognostic score, castration-resistant prostate cancer, prostate-specific antigen, testosterone, inflammation

## Abstract

**Simple Summary:**

Prostate cancer is one of the most prevalent cancers in men. Prostate cancer is characterized by an early response to hormonal therapy and prostate-specific antigen (PSA) is useful for diagnosis, prognosis, and treatment evaluation. However, if the patient becomes resistant to treat and develops castration-resistant prostate cancer (CRPC), it is difficult to predict prognosis and evaluate response to treatment using PSA alone. In this study, we found that the high-sensitivity modified Glasgow prognostic score (Hs-mGPS), an inflammatory response score, is a more powerful prognostic factor for CRPC than the modified Glasgow prognostic score (mGPS) previously studied. Furthermore, we suggest that risk classification using Hs-mGPS, PSA, and testosterone (TST) may be a useful tool to predict the prognosis of late staged CRPC.

**Abstract:**

The Glasgow prognostic score, a marker of systemic inflammation, is associated with clinical outcomes in different cancers including prostate cancer. However, there is no evidence for the relationship between the high-sensitivity modified Glasgow prognostic score (Hs-mGPS) in prostate cancer and its prognosis. This study aimed to investigate the prognostic significance of Hs-mGPS in castration-resistant prostate cancer (CRPC) treated with docetaxel. We retrospectively analyzed clinical datasets from 131 CRPC patients who received docetaxel treatment at Chiba University Hospital and a related hospital. Clinical factors including Hs-mGPS before docetaxel treatment were evaluated according to overall survival. The numbers of patients with Hs-mGPS of 0, 1, and 2 were 88, 30, and 13, respectively. The median prostate-specific antigen (PSA) level was 28.9 ng/mL. The median testosterone level was 13.0 ng/dL. The percentages of bone and visceral metastases were 80.8% and 10.2%, respectively. For overall survival, Hs-mGPS ≥ 1 (hazard ratio of 2.41; *p* = 0.0048), testosterone ≥ 13.0 ng/dL (hazard ratio of 2.23; *p* = 0.0117), and PSA ≥ 28.9 ng/mL (hazard ratio of 2.36; *p* = 0.0097) were significant poor prognostic factors in the multivariate analysis. The results of the two-group analysis showed that a higher Hs-mGPS was associated with high PSA, alkaline phosphatase, and testosterone levels. The median testosterone levels for Hs-mGPS of 0, 1, and 2 were 9.0, 16.5, and 23.0, respectively. Based on the multivariate analysis, we created a combined score with three prognostic factors: Hs-mGPS, testosterone, and PSA. The low-risk group (score of 0–1) showed a significantly longer overall survival compared to the intermediate-risk (score of 2–3) and high-risk (score of 4) groups (*p* < 0.0001). Our results demonstrated that an elevated Hs-mGPS was an independent prognostic factor in CRPC patients treated with docetaxel therapy. Risk classification based on Hs-mGPS, testosterone, and PSA may be useful in predicting the prognosis of CRPC patients.

## 1. Introduction

Worldwide, prostate cancer is the second most common cancer in men, with 1.3 million people diagnosed and 360,000 deaths every year according to a 2018 report. [1]. Androgen deprivation therapy (ADT) is the standard approach to the treatment for locally advanced or metastatic prostate cancer. ADT has a good oncology efficacy for hormone-sensitive prostate cancer (HSPC), but most patients acquire resistance to ADT and will develop castration-resistant prostate cancer (CRPC) [2]. Docetaxel treatment was established for CRPC in 2004 [3,4]. Though other novel therapeutic agents, such as androgen receptor axis-targeting (ARAT), have been developed [5,6], docetaxel is now used as one of the first-line treatments for metastatic hormone-sensitive prostate cancer (mHSPC) with high-volume tumors and metastatic CRPC [7,8,9].

Several studies have revealed that inflammation factors in CRPC provide prognostic information. For example, interleukin (IL)-6 was found to induce the drug resistance of prostate cancer by activating the androgen receptor (AR), and IL-4 was found to be associated with the development of CRPC by regulating coactivators of AR such as The nuclear factor-kappaB (NF-κB) [10,11]. Furthermore, the C-X-C motif chemokine receptor (CXCR) family, which is called CXC chemokine, also plays an important role in the development of CRPC by adjusting inflammation pathways [12,13,14]. Treatment-resistant in CRPC could be caused by a signal response through some inflammatory pathways. Therefore, inflammatory conditions in CRPC patients may be prognostic factors.

The Glasgow prognostic score (GPS), the modified GPS (mGPS), and the high-sensitivity modified GPS (Hs-mGPS) are inflammatory biomarkers that are calculated by values derived from levels of serum albumin and C-reactive protein (CRP) [15,16,17]. GPS and mGPS were previously reported, and they have the same cutoff value (>1.0 mg/dL for CRP and <3.5 g/dL for albumin) [15,16]. Later, Proctor et al. changed the mGPS and reported Hs-mGPS, which has a high-sensitivity cutoff value for CRP (>0.3 mg/dL for CRP and <3.5 g/dL for albumin) [17]. Previous studies have assessed the association between GPS (or mGPS) and survival outcomes in patients with prostate cancer [18,19,20]. However, there is no evidence for the relationship between Hs-mGPS in prostate cancer and its prognosis. Therefore, it would be very interesting to see how well Hs-mGPS predicts the prognosis of CRPC patients.

The aim of the present study was to determine the importance of predicting the prognosis of the Hs-mGPS in CRPC treated with docetaxel.

## 2. Materials and Methods

### 2.1. Study Population and Clinical Variables

The present study was approved by the institutional review board (approval number 2252). The clinical data of 131 patients treated with docetaxel for CRPC at Chiba University Hospital and Funabashi City Medical Center between 2005 and 2019 were retrospectively reviewed. As a first-line hormonal treatment for HSPC, all patients received combined androgen blockade (CAB) therapy.

We reviewed clinical and pathological variables on age, body mass index (BMI), clinical tumor (T) stage, Gleason score, bone metastasis, and visceral metastasis. Blood samples were collected within 4 weeks of the start date of docetaxel treatment, and the results were evaluated. The factors used in the analysis were prostate-specific antigen (PSA), lactate dehydrogenase (LDH), alkaline phosphatase (ALP), testosterone, total protein (TP), total cholesterol (T-CHO), hemoglobin, platelet, and the Hs-mGPS. We used the Architect Testosterone II immunoassay (Abbot Diagnostics, Lake Forest, IL, USA) for the measurement of serum testosterone levels. The prognostic importance of the Hs-mGPS and other clinical factors were assessed for overall survival (OS) and progression-free survival (PFS).

### 2.2. High-Sensitivity Modified Glasgow Prognostic Score

In the present study, we selected Hs-mGPS, an optimized version of the with a sensitivity CRP cut-off point of 0.3. The optimum cut-off for CRP was 0.3 when we analyzed the area under the curve (AUC) calculated from the receiver operating characteristic curve in this study (Figure S1). We calculated the Hs-mGPS, which is constructed in Table 1. Patients with both CRP > 0.3 mg/dL and albumin < 3.5 g/dL were scored as Hs-mGPS_2. Patients with CRP > 0.3 mg/dL and albumin ≥ 3.5 g/dL were scored as Hs-mGPS_1. Patients with CRP < 0.3 mg/dL were scored as Hs-mGPS_0, regardless of the albumin value.

### 2.3. Docetaxel Treatment

Patients were treated with ADT plus docetaxel. In this study, daily prednisolone medication was not required for docetaxel treatment. The dose of docetaxel was 75 mg per square meter of body-surface area administered intravenously every 3–4 weeks. The dose modification of docetaxel was allowed at the discretion of the investigator, and the docetaxel dose was reduced to 55–65 mg/m^2^. Clinicians adjusted the dosage according to the deterioration of performance status and the risk of febrile neutropenia.

### 2.4. Endpoints and Definition of Progression

Overall survival was defined as the number of days from the date of docetaxel initiation to death, regardless of the cause of death. Progression-free survival was defined as the number of days from the date of docetaxel initiation to PSA progression, radiographic progression, or death, regardless of the cause of death. We defined progression as the presence of either PSA progression or radiological progression. PSA progression was decided by the definition of The Prostate Cancer Clinical Trials Working Group 2 (PCWG2) [21]: if there was PSA increase of >2 ng/mL above the nadir or >25% of the nadir, it had to be confirmed by a second PSA test at least 3 weeks later or when the patient’s clinician decided to discontinue docetaxel treatment due to an elevated PSA level. The radiographic progression of bone metastasis was decided by PCWG2 criteria with bone scanning [21]. The progression of lesions of viscera and lymph nodes were determined according to the definition of Response Evaluation Criteria in Solid Tumors (RESIST) version 1.1 with computed tomography (CT) or magnetic resonance imaging (MRI) [22].

### 2.5. Statistical Analysis

We selected the median of continuous variables as the cutoff value when analyzing the prognostic value of each clinical factor. To evaluate the effect of clinical variables on OS and PFS, univariate and multivariate Cox proportional hazard models were analyzed. Hazard ratios and 95% confidence intervals were derived.

We analyzed survival outcomes (OS and PFS) using the Kaplan–Meier method and performed a log-rank test to compare these estimates. The association between Hs-mGPS ≥ 1 and other clinical variables was tested using the chi-squared test and Wilcoxon’s signed-rank test. All tests were 2-sided, and a *p* < 0.05 was considered significant. We used the JMP statistical software for statistical analysis; JMP 11.0.0 (SAS Institute, Cary, NC, USA).

## 3. Results

### 3.1. Patients Characteristics

Table 2 lists the clinical characteristics of the 131 patients included in this study. The median follow-up duration was 21.1 months, and 65 patients (50.0%) died. The Hs-mGPS was 0, 1, and 2 for 88, 30, and 13 patients, respectively. The median age of the patients was 72.0 years, and the median PSA and testosterone (TST) levels from blood data at the start of docetaxel treatment were 28.9 ng/mL and 13.0 ng/dL, respectively. Of the 131 patients, 6.1% and 13.0% received enzalutamide and abiraterone before docetaxel treatment, respectively. Figure S2 shows the distribution of CRP, and the median CRP level was 0.15 mg/dL (interquartile range (IQR): 0.10–0.50 mg/dL).

### 3.2. Survival Analysis Using the Kaplan–Meier Methods in CRPC Patients According to Hs-mGPS

**Figure 1:**
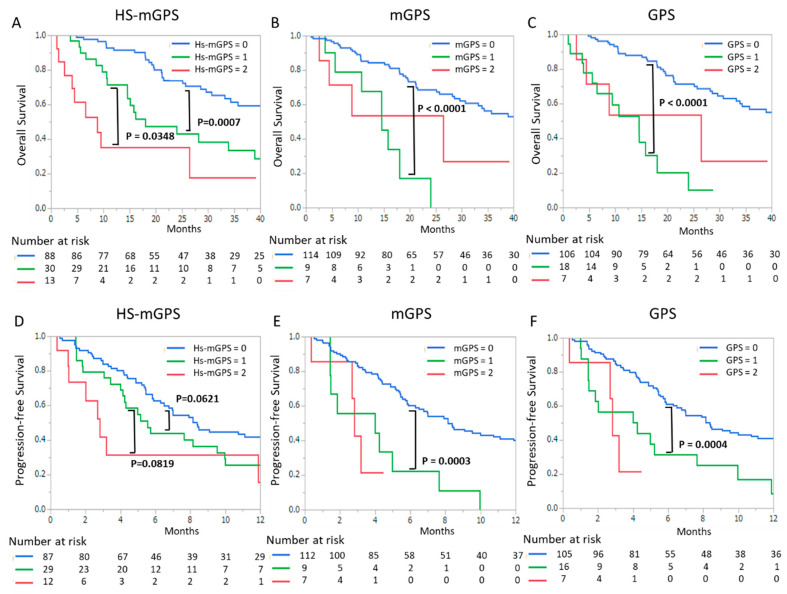
Kaplan–Meier curves for the high-sensitivity modified Glasgow prognostic score (HS-mGPS) (**A**,**D**), the modified Glasgow prognostic score (mGPS) (**B**,**E**), and the Glasgow prognostic score (GPS) (**C**,**F**). (**A**–**C**) and (**D**–**F**) indicate overall survival and progression-free survival in docetaxel treatment, respectively. *p*-values were calculated by the log-rank test.

Figure 1 presents the Kaplan–Meier curves for OS (Figure 1A–C) and PFS (Figure 1D–F) with Hs-mGPS (Figure 1A,D), mGPS (Figure 1B,E), and GPS (Figure 1C,F). The median OS was 21.1 months in all patients. The median OS times of Hs-mGPS_2, 1, and 0 were 8.8, 18.1, and 51.8 months, respectively. A high Hs-mGPS was significantly associated with a poor prognosis; the OS between Hs-mGPS_2 and 1 and between Hs-mGPS_1 and 0 were significantly different (*p* = 0.0007 and *p* = 0.0348, respectively). In addition, PFS was shorter in the high Hs-mGPS group than in the low Hs-mGPS group (the median OS values of Hs-mGPS_2, 1, and 0 were 2.7, 5.1, and 6.8 months, respectively). On the other hand, in the analysis on mGPS, there was a significant difference in the survival (OS and PFS) of mGPS_1 and 0 (*p* < 0.0001 and *p* = 0.0003, respectively) but not mGPS_2 and 1 (*p* = 0.4374 and *p* = 0.7018, respectively). Similar to the analysis of mGPS, the analysis of GPS showed no significant difference in survival outcomes between scores of 2 and 1.

### 3.3. The Predictive Role of the Hs-mGPS in the Survival Outcome of CRPC Patients

Table 3 presents the results of univariate and multivariate Cox proportional hazard analyses for OS. The univariate analysis showed that bone metastasis (*p* = 0.0399), PSA (*p* < 0.0001), alkaline phosphatase (*p* = 0.0174), testosterone (*p* = 0.0007), and Hs-mGPS (*p* < 0.0001) were significant prognostic factors associated with OS. The multivariate analysis showed that PSA (*p* = 0.0097), testosterone (*p* = 0.0117), and Hs-mGPS (*p* = 0.0048) were significantly prognostic for OS. Table 3 also presents the results of univariate and multivariate Cox proportional hazard analyses for PFS. The univariate analysis showed that bone metastasis (*p* = 0.0047), PSA (*p* < 0.0001), alkaline phosphatase (*p* = 0.0042), testosterone (*p* = 0.0049), and Hs-mGPS (*p* = 0.0078) were significant prognostic factors associated with PFS. The multivariate analysis showed that Hs-mGPS (*p* = 0.0945) was not significantly prognostic for PFS.

**Figure 2:**
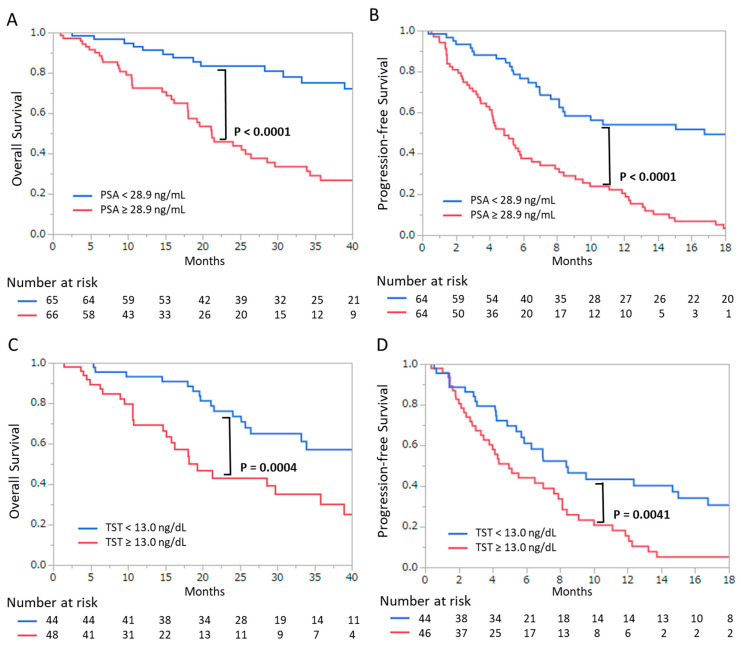
Kaplan–Meier curves for (**A**) overall survival and (**B**) progression-free survival according to prostate-specific antigen levels (<28.9 ng/mL or ≥28.9 ng/mL). Kaplan–Meier curves for (**C**) overall survival and (**D**) progression-free survival according to serum testosterone levels (<13 ng/dL or ≥13 ng/dL). *p*-values were calculated by the log-rank test.

Figure 2 presents the Kaplan–Meier curves for survival outcomes according to PSA and TST values. OS (Figure 2A) and PFS (Figure 2B) were significantly shorter in the high PSA group than in the low PSA group (*p* < 0.0001 and *p* < 0.0001, respectively). Additionally, OS (Figure 2A) and PFS (Figure 2B) were also significantly shorter in the high TST group than in the low TST group (*p* = 0.0004 and *p* = 0.0041, respectively).

### 3.4. The Characteristics of the High Hs-mGPS Patients

Table 4 shows the clinical characteristics associated with Hs-mGPS, and two groups (Hs-mGPS < 1 vs. Hs-mGPS ≥ 1) were compared. The Hs-mGPS ≥ 1 group showed significantly higher PSA, testosterone, and alkaline phosphatase levels (*p* = 0.0271, *p* = 0.0182, and *p* = 0.0388, respectively) and lower hemoglobin levels (*p* = 0.0033). Table 4 also shows the treatment before and after docetaxel. There was no significant difference in the rate of pre-ARATs between the two groups (*p* = 0.9221). On the other hand, there was a difference in post-docetaxel treatment, although it was not statistically significant (*p* = 0.1149). To eliminate the bias of sequential treatment after docetaxel, the survival analysis was performed for 92 patients who received only best supportive care (BSC). Figure S3 shows that a high Hs-mGPS was significantly associated with a poor prognosis in patients who received the same sequential treatment (BSC).

We focused on independent prognostic factors and added analysis. Table S1 shows PSA and TST levels according to the Hs-mGPS. Though significant differences were not clear, TST values tended to increase as the Hs-mGPS increased; the median TST levels with Hs-mGPS_0, 1, and 2 were 9.0, 16.5, and 23.0, respectively.

### 3.5. Combined Score and Risk Classification

**Figure 3:**
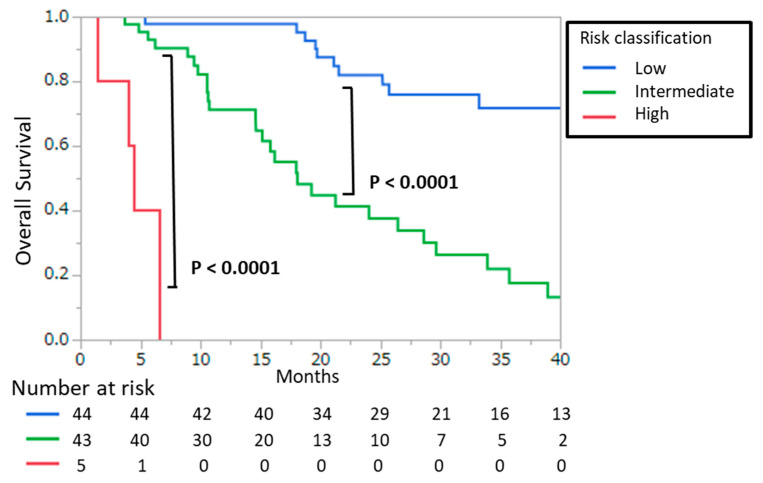
Kaplan–Meier curves for overall survival according to risk classification. Risk classification was determined based on a combined score, which was the sum of the three prognostic scores (see Table 5). High-, intermediate-, and low-risk were defined as combined scores of 4, 2–3, and 0–1, respectively. *p*-values were calculated by the log-rank test.

Table 5 shows the risk classification using a combined score. Based on the significant prognostic factors in the multivariate analysis, the combined score was calculated with the Hs-mGPS (score of 0–2), PSA score (score of 0–1), and TST score (score of 0–1). A patient with PSA > 28.9 mg/mL was scored as 1, and a patient with TST > 13.0 ng/dL was scored as 1. To determine the prognostic power of the combined score, we divided patients into three risk groups: the low risk group had 0–1 risk scores, the intermediate group had 2–3 risk scores, and the high risk group had a 4 risk score. Associations between risk classification and OS were evaluated using the Kaplan–Meier method (Figure 3). The results of the log-rank test revealed a statistically significant difference between the groups (low vs. intermediate-risk: *p* < 0.0001; intermediate vs. high-risk: *p* < 0.0001). In the high-, intermediate-, and low-risk groups, the median OS values were 5.4, 21.2, and 58.3 months, respectively.

**Figure 4:**
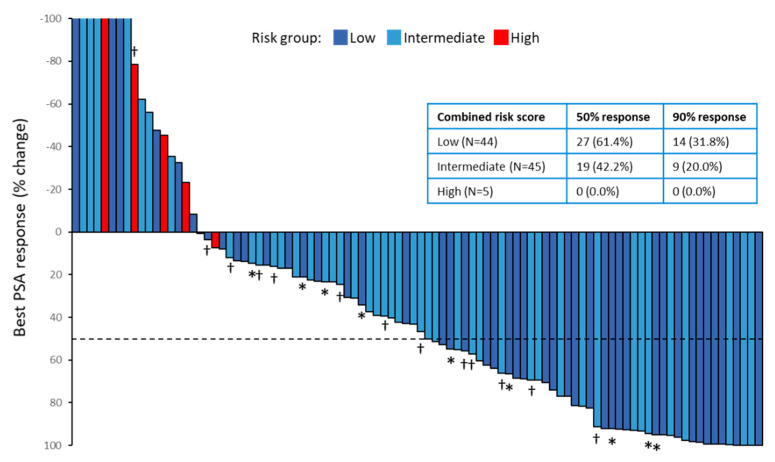
The waterfall plot for the largest prostate-specific antigen (PSA) declines from baseline. Red, light blue, and deep blue bars indicate high-risk, intermediate-risk, and low-risk, respectively. The dotted line shows the threshold for a 50% PSA response (≥50% reduction in PSA level from baseline). Asterisks indicate patients who had previously received abiraterone or enzalutamide, and daggers indicate patients who had previously received abiraterone and enzalutamide. The table in the figure shows the percentage of the 50% response and the 90% response by risk group.

Figure 4 shows the waterfall plot of the best PSA responses to docetaxel treatment according to the risk classification. In all patients, the overall proportion of patients who had a 50% PSA response was 48.9%. The 50% PSA response rate among the low-risk group was 61.4%, the rate among the intermediate-risk group was 42.2%, and the rate among the high-risk group was 0.0%. The 90% PSA response rates among the low-risk and intermediate-risk group were 31.8% and 20.0%, respectively. Our study included 25 patients who had previously received abiraterone or enzalutamide before docetaxel treatment.A two-arm analysis was performed to analyze whether pretreatment with anti-androgen agents affects the response of the Comined Score and PSA. (Table S2). No association between the pretreatment of anti-androgen (enzalutamide or abiraterone) and the combined score or PSA response was statistically significant (*p* = 0.3099 and *p* = 0.7088, respectively).

## 4. Discussion

To our knowledge, this study shows the first evidence of the clinical significance of the Hs-mGPS in CRPC patients receiving docetaxel treatment. A high Hs-mGPS is highly associated with worse outcomes (OS and PFS) in CRPC patients receiving docetaxel treatment. We also found that the prognostic power of Hs-mGPS was superior to that of the mGPS and GPS. Furthermore, the present study showed that a serum prostate-specific antigen level of 28 ng/mL and a serum testosterone level of 13 ng/dL were found to be significant prognostic factors. We thus constructed a combined score using these three independent risk factors (Hs-mGPS, TST, and PSA) and classified three risk groups. Our results showed the greater prognostic significance of this risk classification than that of single Hs-mGPS.

The importance of evaluating the mGPS in prostate cancer has been previously shown. For HSPC, Shafique et al. reported that a high mGPS had significantly poorer survival outcomes in HSPC treated with ADT [18,23]. They showed that the prognostic importance of mGPS appeared to be superior to the neutrophil-to-lymphocyte ratio (NLR). On the other hand, for CRPC, two studies have evaluated the significance of mGPS. In 2013, Linton et al. reported that the mGPS in CRPC was a significant predictor for survival outcomes. Their data showed the mGPS_2 group had a significantly shorter OS than the mGPS_0 group, but the difference between the mGPS_1 and mGPS_0 groups was not clear [19]. In 2019, Kremuser et al. also evaluated the prognostic role of the mGPS in CRPC. They showed that the mGPS predicted disease progression but not OS [20]. Previous reports have suggested that mGPS is a prognostic factor in CRPC patients. However, it was uncertain whether mGPS is the prognostic factor for OS or not in CRPC.

The Hs-mGPS was established by Proctor et al. in 2013 [17]. They modified the threshold of CRP to enhance the prognostic value of the mGPS. Their results suggested a serum CRP level of 0.3 mg/dL is a potentially significant threshold and improves the prognostic importance of the mGPS. The prognostic superiority of the Hs-mGPS over mGPS has been demonstrated in many series of nonurological malignancies such as neck and esophageal cancer [24,25]. For example, in 2020, Tao Hou et al. reported that the Hs-mGPS was significantly associated with overall survival and disease-free survival in patients with soft-tissue sarcoma who underwent radical surgery [26]. Despite the important prognostic role of the Hs-mGPS in various malignancies, there has been no study that evaluates the association between Hs-mGPS and its prognosis in prostate cancer. We evaluated the association between Hs-mGPS and OS in CRPC for the first time and showed that the Hs-mGPS is superior to the mGPS as a strong prognostic factor.

The short OS and PFS in the high Hs-mGPS group suggested that the response to docetaxel is poor in patients with high inflammatory status. This may be due to the enhancement of docetaxel resistance by inflammatory molecules. Cytokines (IL-6 and IL-8) have been reported to stimulate prostate cancer cell proliferation in an autocrine and paracrine manner, as well as to inhibit apoptotic pathways [27,28]. Therefore, these inflammatory molecules may be involved in docetaxel resistance. C-C motif chemokine 2(CCL2) are upregulated by docetaxel treatment, and CCL2 has been implicated in docetaxel resistance by stimulating the extracellular signal-related kinase (ERK)/ mitogen-activated protein kinase (MAPK)and phosphatidylinositol-3 kinase (PI3K)/ protein kinase B (Akt) signaling pathways [29,30,31].

We showed that a high Hs-mGPS, an inflammation marker, was associated with a high testosterone level. Though this association had not been clarified, we speculated that a high Hs-mGPS was related to some inflammation factors that cause the synthesis of testosterone. Transforming growth factor-beta (TGF-β) and insulin-like growth factor (IGF)-β could regulate the synthesis of the intratumoral androgen [32]. IL-6 and IGF2 could also activate the synthesis of intratumoral androgen via steroidogenic enzymes [32,33]. These reports suggested that inflammation factors such as interleukin may activate intratumoral testosterone synthesis in CRPC. A previous report suggests that testosterone attenuates the effect of taxane anticancer drugs. This suggests that intratumoral testosterone synthesis may be associated with treatment resistance and poor prognosis [34]. Furthermore, we also showed that a high Hs-mGPS was associated with high PSA, ALP, and LDH levels. It is known that these biochemical data are associated with tumor volume. The Hs-mGPS may be associated with tumor volume, which reflects treatment resistance and poor prognosis.

Our laboratory has reported a good prognosis for HSPC patients who achieve a testosterone level of less than 20 ng/dL with ADT treatment [35]. However, in this study, the median testosterone level in the high Hs-mGPS group was 23 ng/dL in the two-arm analysis (Table 4), suggesting that the level of castration may be insufficient. An insufficient castration level may be associated with docetaxel resistance. Abiraterone is known to be a potent testosterone reducer in CRPC patients [36]. Our laboratory showed that CRPC patients with high testosterone levels also have a good response to enzalutamide [37]. In patients with high testosterone levels (>20 ng/dL), stronger castration with abiraterone or enzalutamide before docetaxel may have a better prognosis.

Currently, the most clinically used prognostic factors for CRPC are PSA and the presence of visceral metastasis. The Hs-mGPS was still a significant independent prognostic factor in this study, even after multivariate analysis including these factors. The clinical usefulness of the Hs-GPS is that it can be easily measured by biochemical tests, and it can improve prognosis prediction when evaluated together with other tests such as PSA. To strengthen the prognostic power of the Hs-mGPS, a combined score was created by adding independent prognostic factors (PSA and testosterone) to create a risk classification. Though Kremuser et al. created a nomogram that is calculated by the mGPS, NLR, PSA, etc., they did not evaluate testosterone [20]. For the first time, we performed a risk classification of the Hs-mGPS including testosterone, and we significantly improved the prognostic ability of the Hs-mGPS. Furthermore, we showed that the high-risk group had a lower PSA response in CRPC patients treated with docetaxel. This suggested that the high-risk group was associated with resistance to docetaxel treatment, which may have led to a poor prognosis.

One of the limitations of the current study was its retrospective study design, so there might have been some selection bias. Performing a prospective and large scale study is required. Second, the small number of patients limited the power of the study to identify fewer potential effects. Third, We could not analyze the association between the NLR and prognostic effect because we did not have enough data on neutrophil and lymphocyte counts. It has been known that an elevated NLR is associated with worse survival outcomes in patients with many cancers. In the future, it would be of great interest to compare the prognostic impact of the Hs-mGPS with that of the NLR in CRPC patients. Fourth, some patients received various post-docetaxel therapy including enzalutamide, abiraterone acetate, and cabazitaxel. Post-docetaxel therapy could influence survival outcomes. Fifth, docetaxel dose adjustments were performed at the discretion of the investigators, and there were no clear criteria. The degree of docetaxel reduction might make a difference in the effectiveness of chemotherapy.

## 5. Conclusions

In this study, we demonstrated that an elevated Hs-mGPS was an independent prognostic factor in CRPC patients who received docetaxel treatment. We suggested that assessing the Hs-mGPS, the prostate-specific antigen level, and the testosterone level may be a more powerful predictor of CRPC prognosis.

## Figures and Tables

**Table 1:** The development of the Glasgow prognostic score.

**Glasgow Prognostic Score**	**Score**
C-reactive protein < 1.0 mg/dL and albumin ≥ 3.5 g/dL	0
C-reactive protein ≥ 1.0 mg/dL or albumin < 3.5 g/dL	1
C-reactive protein ≥ 1.0 mg/dL and albumin < 3.5 g/dL	2
**Modified Glasgow prognostic score**	**Score**
C-reactive protein < 1.0 mg/dL and albumin ≥ 3.5 g/dL	0
C-reactive protein ≥ 1.0 mg/dL and albumin ≥ 3.5 g/dL	1
C-reactive protein ≥ 1.0 mg/dL and albumin < 3.5 g/dL	2
**High-sensitivity modified Glasgow prognostic score**	**Score**
C-reactive protein < 0.3 mg/dL and albumin ≥ 3.5 g/dL	0
C-reactive protein ≥ 0.3 mg/dL and albumin ≥ 3.5 g/dL	1
C-reactive protein ≥ 0.3 mg/dL and albumin < 3.5 g/dL	2

**Table 2:** Clinical characteristics of CRPC patients in the present study (*n* = 131). IQR: interquartile range.

Characteristics	Value: Median (IQR) or *n* (%)
Age (years)	72.0 (67.0–77.0)
BMI (kg/m^2^)	23.6 (21.6–25.8)
Follow-up duration (months)	21.1 (10.6–38.9)
ADT duration before docetaxel (months)	35.5 (18.2–61.0)
Gleason score (*n* = 88)	
5–7	17 (19.2%)
8–10	59 (80.9%)
Metastasis	
Bone metastasis	101 (80.8%)
Visceral metastasis	12 (10.2%)
PSA (ng/mL)	28.9 (4.6–73.4)
Testosterone (ng/dL)	13.0 (5.0–23.0)
Lactate dehydrogenase (IU/L)	212.0 (183.0–248.5)
Alkaline phosphatase (IU/L)	295.5 (226.5–471.0)
Total protein (g/dL)	6.6 (6.4–6.9)
Albumin (g/dL)	4.1 (3.7–4.3)
Total cholesterol (mg/dL)	216.0 (190.5–241.5)
Hemoglobin (g/dL)	12.5 (11.5–13.2)
Platelet (×10^3^/μL)	2.2 (1.8–2.6)
C-reactive protein (mg/dL)	0.15 (0.10–0.50)
Pre-ARATs	
No	106 (80.9%)
Enzalutamide	8 (6.1%)
Abiraterone	17 (13.0%)

CRPC, castration-resistant prostate cancer; IQR, interquartile range; BMI, body mass index; ADT, androgen deprivation therapy; PSA, prostate-specific antigen; ARAT, androgen receptor axis-targeted therapy.

**Table 3:** Cox proportional hazard analysis for progression-free survival and overall survival.

Overall Survival	Univariate Analysis		Multivariate Analysis	
	HR (95% CI)	*p*	HR (95% CI)	*p*
Age ≥ 73 year	1.05 (0.65–1.71)	0.8177		
BMI ≥ 23.7 kg/m^2^	1.08 (0.55–2.12)	0.8036		
Clinical tumor stage ≥ 3	2.76 (0.37–20.16)	0.3150		
Gleason score ≥ 8	1.20 (0.55–2.60)	0.6419		
Visceral metastasis	2.10 (0.88–5.01)	0.0934		
Bone metastasis	2.08 (1.05–4.13)	0.0399	2.10 (0.78–5.60)	0.1375
PSA ≥ 27.0 ng/mL	3.17 (1.87–5.36)	<0.0001	2.36 (1.23–4.54)	0.0097
Lactate dehydrogenase ≥ 213 IU/L	1.02 (0.62–1.67)	0.9356		
Alkaline phosphatase ≥ 293 IU/L	1.80 (1.11–2.95)	0.0174	1.13 (0.61–2.09)	0.6880
Total protein (g/dL)	0.95 (0.49–1.83)	0.8984		
Total cholesterol (mg/dL)	0.96 (0.48–1.93)	0.9297		
Testosterone ≥ 13.0 ng/dL	2.88 (1.56–5.31)	0.0007	2.23 (1.19–4.16)	0.0017
Hemoglobin (g/dL)	0.65 (0.40–1.08)	0.0923		
Platelet (×10^3^/μL)	1.35 (0.82–2.21)	0.2277		
Hs-mGPS ≥ 1	2.97 (1.81–4.87)	<0.0001	2.41 (1.31–4.46)	0.0048
**Progression-Free Survival**	**Univariate Analysis**		**Multivariate Analysis**	
	**HR (95% CI)**	***p***	**HR (95% CI)**	***p***
Age ≥ 73 year	0.73 (0.49–1.10)	0.1396		
BMI ≥ 23.7 kg/m^2^	1.05 (0.61–1.80)	0.8563		
Clinical tumor stage ≥ 3	3.06 (0.74–12.54	0.1198		
Gleason score ≥ 8	1.72 (0.87–3.41)	0.1168		
Visceral metastasis	1.25 (0.62–2.50)	0.5258		
Bone metastasis	2.34 (1.29–4.22)	0.0047	1.79 (0.84–3.79)	0.1266
PSA ≥ 27.0 ng/mL	3.80 (2.38–6.07)	<0.0001	2.45 (1.32–4.54)	0.0044
Lactate dehydrogenase ≥ 213 IU/L	1.12 (0.74–1.68)	0.5808		
Alkaline phosphatase ≥ 293 IU/L	1.82 (1.20–2.74)	0.0042	0.84 (0.47–1.51)	0.5751
Total protein (g/dL)	0.87 (0.50–1.51)	0.6376		
Total cholesterol (mg/dL)	1.29 (0.72–2.31)	0.3857		
Testosterone ≥13.0 ng/dL	2.01 (1.23–3.25)	0.0049	1.42 (0.84–2.39)	0.1876
Hemoglobin (g/dL)	0.71 (0.47–1.07)	0.1091		
Platelet (×10^3^/μL)	1.38 (0.92–2.07)	0.1182		
Hs-mGPS ≥ 1	1.76 (1.16–2.68)	0.0078	1.55 (0.92–2.59)	0.0945

HR, hazard ratio; CI, confidence interval; BMI, body mass index; PSA, prostate-specific antigen. Hs-mGPS, high-sensitivity modified Glasgow prognostic score.

**Table 4:** Clinical variables and high-sensitivity modified Glasgow prognostic score (1< and ≥1).

Characteristics	Median (IQR) or *n* (%)		*p*
	Hs-mGPS < 1	Hs-mGPS ≥ 1	
Age (years)	72.0 (67.0–76.0)	72.5 (67.3–77.0)	0.7703 ^†^
BMI (kg/m^2^)	23.6 (22.0–25.3)	23.7 (21.4–25.9)	0.9698 ^†^
ADT duration before docetaxel (months)	33.1 (17.8–61.1)	35.6 (18.5–62.0)	0.7109 ^†^
Gleason score ≥ 8 (*n* = 88)	50 (83.3%)	21 (75.0%)	0.3642 ^¶^
Bone metastasis	65 (77.4%)	36 (87.8%)	0.1518 ^¶^
Visceral metastasis	8 (9.8%)	4 (10.8%)	0.8768 ^¶^
PSA (ng/mL)	21.7 (4.1–59.6)	41.9 (14.1–125.6)	0.0271 ^†^
Testosterone (ng/dL)	9.0 (3.0–20.5)	18.0 (10.0–27.0)	0.0182 ^†^
Lactate dehydrogenase (IU/L)	209.0 (179.0–273.2)	229.0 (186.0–288.0)	0.0260 ^†^
Alkaline phosphatase (IU/L)	286.0 (219.0–450.0)	369.0 (240.0–685.0)	0.0388 ^†^
Hemoglobin (g/dL)	12.7 (11.9–13.2)	11.8 (11.0–12.9)	0.0033 ^†^
Platelet (×10^3^/μL)	21.6 (18.7–26.2)	22.4 (18.0–24.9)	0.8561 ^†^
Pre-ARATs			
No	71 (80.7%)	35 (81.4%)	0.9221 ^¶^
Enzalutamide	5 (5.7%)	3 (6.9%)	
Abiraterone	12 (13.6%)	5 (11.6%)	
Post-docetaxel treatment			
No	58 (65.9%)	34 (79.0%)	0.1149 ^¶^
Enzalutamide	10 (11.4%)	6 (13.9%)	
Abiraterone	11 (12.5%)	1 (2.3%)	
Cabazitaxel	9 (10.2%)	2 (4.7%)	

IQR, interquartile range; Hs-mGPS, high-sensitivity modified Glasgow prognostic score; BMI, body mass index; ADT, androgen deprivation therapy; PSA, prostate-specific antigen; ARAT, androgen receptor axis targeted therapy. ^†^ Wilcoxon signed-rank test; ^¶^ chi-square test.

**Table 5:** Combined score and risk classification based on 3 independent prognostic factors.

Prognostic Factors -	Score
Hs-mGPS	
CRP < 0.3 mg/dL and albumin ≥ 3.5 g/dL	0
CRP ≥ 0.3 mg/dL and albumin ≥ 3.5 g/dL	1
CRP ≥ 0.3 mg/dL and albumin < 3.5 g/dL	2
PSA score	
PSA < 28.9 ng/mL	0
PSA ≥ 28.9 ng/mL	1
TST score	
TST < 13.0 ng/dL	0
TST ≥ 13.0 ng/dL	1
**Risk Groups**	Combined score *
Low	0–1
Intermediate	2–3
High	4

Hs-mGPS, high-sensitivity modified Glasgow prognostic score; CRP, C-reactive protein; PSA, prostate-specific antigen; TST, testosterone. * Combined score = Hs-mGPS + PSA score + TST score.

## Data Availability

The data presented in this study are available on request from the corresponding author. The data are not publicly available due to ethical restrictions.

## Supplementary Materials

The following are available online at https://www.mdpi.com/2072-6694/13/4/773/s1, Table S1: Relationships of PSA and testosterone according to Hs-mGPS. Figure S1: Receiver operating characteristic (ROC) curves for C-reactive protein (CRP) to predict death, regardless of the cause of death. Figure S2: Distribution of CRP level at the start of docetaxel therapy. Figure S3: Kaplan-Meier curves for OS according to Hs-mGPS in patients who had received only best supportive care (BSC) after docetaxel. P-values were calculated by the log-rank test., Table S2: Two-group analysis with and without ARAT (abiraterone or enzalutamide) treatment before docetaxel.
