# Supplementary material for: Prognostic Value of High-Sensitivity Modified Glasgow Prognostic Score in Castration-Resistant Prostate Cancer Patients Who Received Docetaxel
Source: Cancers (Basel). 2021 Feb 12;13(4):773. doi: 10.3390/cancers13040773 (PMC7918602; doi:10.3390/cancers13040773)
Supplement: Supplementary file 1 [file cancers-13-00773-s001.pdf]

cancers

Supplementary Material:

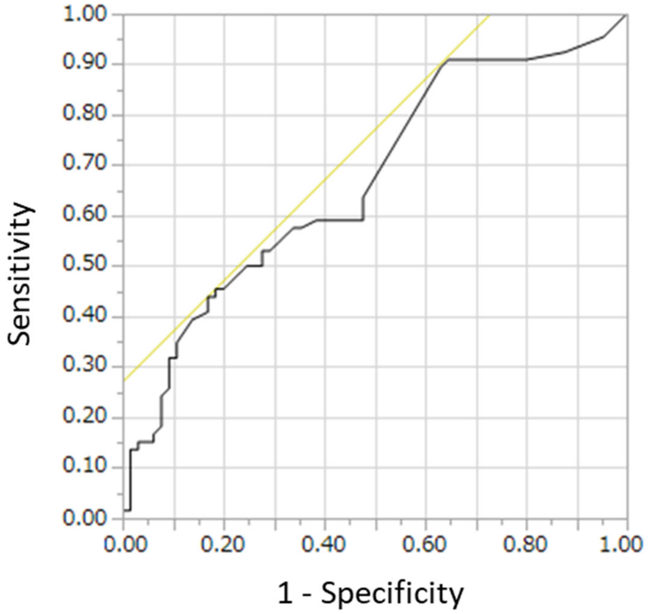

Figure S1. Receiver operating characteristic (ROC) curves for C-reactive protein (CRP) to predict death, regardless of the cause of death. Area under the curve (AUC) of CRP was 0.668 (P = 0.0522).

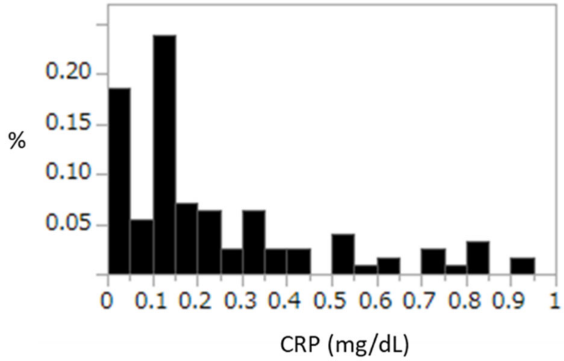

Figure S2. Distribution of CRP level at the start of docetaxel therapy.

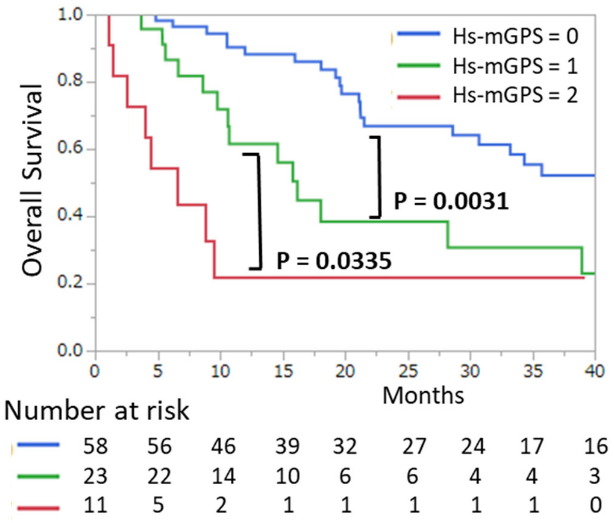

Figure S3. Kaplan-Meier curves for OS according to Hs-mGPS in patients who had received only best supportive care (BSC) after docetaxel. P-values were calculated by the log-rank test.

Table S1. Relationships of PSA and testosterone according to Hs-mGPS.

**Hs-mGPS**	**Median/Average**
	Prostate specific antigen (ng/mL)
0	21.7 / 64.3
1	45.1 / 67.5
2	41.9 / 498.7
	Testosterone (ng/dL)
0	9.0 / 13.4
1	16.5 / 17.1
2	23.0 / 21.5

PSA, prostate specific antigen; TST, testosterone, Hs-mGPS, high-sensitivity modified Glasgow prognostic score

Table S2. Two-group analysis with and without ARAT (abiraterone or enzalutamide) treatment before docetaxel.

	**ARAT (+) N = 22**	**ARAT (-) N = 72**	**P value**
Combined score*			
2-4	14 (63.6%)	37 (51.4%)	0.3099¶
0-1	8 (36.4%)	35 (48.6%)	
PSA response			
≥ 50% response	10 (45.5%)	36 (50.0%)	0.7088¶
< 50% response	12 (54.5%)	36 (50.0%)	

PSA, prostate-specific antigen; ARAT, androgen receptor axis targeted therapy, ¶ chi-square test, * The combined score was calculated with the high sensitivity modified Glasgow prognostic score (score 0-2), PSA score (score 0-1), and testosterone score (score 0-1). A patient with PSA > 28.9 mg/mL was scored as 1, and a patient with TST > 13.0 ng/dL was scored as 1.
